# Correlation of Work Fatigue and Mental Workload in Train Drivers: A Cross-sectional Study

**DOI:** 10.34172/jrhs.2023.135

**Published:** 2023-12-29

**Authors:** Narmin Hassanzadeh-Rangi, Hamed Jalilian, Ali-Asghar Farshad, Yahya Khosravi

**Affiliations:** ^1^Department of Occupational Health and Safety Engineering, School of Health, Alborz University of Medical Sciences, Karaj, Iran; ^2^Research Center for Health, Safety, and Environment, Alborz University of Medical Sciences, Karaj, Iran; ^3^Postdoctoral Researcher at the Swiss Tropical and Public Health Institute, Basel, Swtizerlad; ^4^Department of Occupational Health and Safety Engineering, School of Health, Qom University of Medical Sciences, Qom, Iran; ^5^Occupational Health Research Center, Iran University of Medical Sciences, Tehran, Iran; ^6^Non-Communicable Diseases Research Center, Alborz University of Medical Sciences, Karaj, Iran

**Keywords:** Work fatigue, Mental workload, Train driver, Metro

## Abstract

**Background:** Evidence suggests that train drivers experience a high level of fatigue and mental workload. The present study aimed to assess overall, physical, and mental fatigue levels and their correlations with the mental workload in the metro train operation.

**Study Design:** A cross-sectional study.

**Methods:** This study was conducted on all 1194 train drivers in the Tehran Metro. The train drivers completed the Samn-Perelli Fatigue Scale and the Fatigue Assessment Scales at the beginning and end of the shift. In addition, they completed the National Aeronautics and Space Administration Task Load Index in the middle and at the end of the shift. Correlation and regression analyses were performed on the data to test the study hypothesis.

**Results:** Overall, physical, and mental fatigue levels increased significantly at the end of the shift compared to the onset of the shift (*P*<0.001). The mental workload and related dimensions were significantly increased at the end of the shift compared to the middle of the shift (*P*<0.001). Mental demand was the most important workload problem among the train drivers. The highest correlation was found between overall workload and time pressure (R=0.68, *P*<0.001).

**Conclusion:** The mental workload had a significant correlation with work fatigue in the train drivers. Control measures should be focused on the mental workload and related dimensions, especially mental demand and time pressure.

## Background

 Metro transportation is a complex human-machine system with different levels of automation. The automation levels are defined according to which basic functions of train operation are the responsibility of both the driver and the system itself. The International Association of Public Transport and the international standard IEC 62290 classify trains based on their grades of automation (GoA), including GoA1, GoA2, GoA3, and GoA4. GoA1 trains have automatic train operation (ATP) but are manually driven. GoA2 trains have automated train operation (ATO) with a driver in the cab. The driver is responsible for critical functions such as closing doors and starting the train. GoA3 trains also have ATO but have a train attendant in the passenger coach, whose important duty is monitoring the safe closing of doors. GoA4 trains are capable of unattended train operation.^1^ In GoA1, the operation of the train is based on the ATP system and train driver activities. A train driver directs the train on the track, stops the train at the stations, controls the doors, and responds to emergencies.^2^ Thus, at this level, the train driver’s duty is a set of actions, including observation of the surrounding environment, maintenance of situation awareness, automated cognitive processes, recognition, dynamic decision-making, communication, and action-reaction to drive the train safely. Furthermore, they must integrate information from many sources; for example, trackside signals, rulebooks, timetables, and trackside environment.^3,4^ Numerous studies have shown that train drivers experience high mental work demands compared with other railway employees.^5^ Train drivers require functional both short- and long-term memory. Moreover, the train drivers suffer from heavy mental needs, including demonstration, divided attention, and sustained attention.^6^

 Human cognitive abilities and limitations play an important role in the majority of industrial accidents and driving crashes.^7-9^ Fatigue, especially mental one, is generally inevitable for employees. Fatigue usually results in work-related damage, decreased productivity, performance loss, and critical human errors.^10^ Therefore, management of mental fatigue is essential from the point of view of controlling work risk, productivity, and job well-being. Multiple studies have indicated that different levels of mental workload can induce physical and mental fatigue in train drivers.^11^ There is no single agreed definition of fatigue, but generally, fatigue is considered a state of perceived weariness resulting from prolonged working, a heavy workload, insufficient rest, and inadequate sleep.^12^ Several studies have investigated fatigue in railway industries. Catelani et al reported significant fatigue among train drivers, especially at the end of shift.^13^ In a Chinese railway study, the train drivers expressed a high level of fatigue influenced by working overtime and mental workload.^14^ Dorrian et al revealed that driving with high fatigue becomes less well-planned, resulting in reduced efficiency and safety. Additionally, the level of mental fatigue experienced by train drivers may vary depending on the train and the occurrence of unusual conditions. This mental fatigue can lead to diminished performance by the driver and a decrease in safety due to an increase in traffic violations.^15^ Fatigue can impair the physical performance of humans.^16^ Tsao et al found a high level of physical fatigue in Chinese railway drivers.^14^ Baysari et al concluded that incidents caused by “human failure” were associated with an adverse physiological state and physical fatigue.^17^ The Federal Railroad Administration (FRA) reported that human factors are responsible for nearly 40% of all train accidents, and the FRA confirmed that fatigue plays a role in approximately one out of four of those accidents.^18^

 Many studies have documented that mental fatigue in train drivers is associated with various factors, such as shift work,^19^ monotony,^20^ and workload.^11,21^ While different dimensions of fatigue have been studied in conventional train operations, there has been less focus on mental workload and more emphasis on sleep problems. Limited studies are available on the fatigue and mental workload of metro train operations, and there are a few experimental studies in this regard.^4,22^ Previous research has emphasized the significance of fatigue in the railway industry,^12,23^ but the relationship between fatigue and the mental workload of train drivers remains unclear and has not been thoroughly investigated. This research gap is particularly pronounced in the field of subway transportation. Therefore, the objective of this study was to focus on the overall fatigue, physical fatigue, and mental fatigue experienced by a large sample of metro train drivers and to examine their correlation with mental workload and other potential factors.

## Methods

###  Participants and Study Procedure

 The cross-sectional study was conducted on all 1194 drivers from five metro lines during the spring and summer of 2019. They registered as drivers in the Tehran Subway Company. Researchers in the master station of each line explained the study objectives to the drivers and distributed the informed consent form and demographic, fatigue, and mental workload questionnaires among them. A complete set of instructions for all questionnaires was presented to the drivers. Then, they were requested to fill out the demographic and fatigue questionnaires. The drivers were asked to complete the mental workload questionnaire during break times (middle time of shift work) and the fatigue and mental workload questionnaires at the end of the shift in the presence of researchers. The inclusion criteria were 1-year work experience in train driving and physical and mental health. On the other hand, the exclusion criteria were a recruit or apprentice, chronic or acute disorders, and discontentment to participate in the study.

 Out of 1194 distributed questionnaires, 896 questionnaires were completed, and 33 drivers were excluded because of the exclusion criteria. Finally, 863 questionnaires were included in the final analysis. All study methods, including human participation, followed the guidelines and regulations approved by the Iran University of Medical Sciences. These guidelines are based on the general principles for ethics in medical science research in the Islamic Republic of Iran and the Declaration of Helsinki. Written informed consent was obtained from all participants before they took part in this study.

###  Measures

 A short demographic questionnaire included age, stature, weight, educational level, shift work, work experience, and the like (Table 1).

**Table 1:** Demographic and occupational characteristics of participants (N = 863)

**Continuous variables**	**Mean**	**SD**
Age (y)	29.73	4.00
Work experience (y)	6.18	3.34
Night sleep (y)	7.24	3.44
**Categorical variables**	**Number**	**Percent**
Shift work		
Morning	576	66.8
Afternoon	248	28.7
Night	39	4.5
Second job		
Yes	47	5.5
No	816	94.5
Education level		
Diploma	115	13.3
Associate degree	449	51.9
B.Sc. or M.Sc.	299	34.7
Home to work distance (h)		
< 1	579	67.1
1-2	273	31.6
> 2	11	1.3
Smoking		
Yes	65	7.5
No	798	92.5

 Train drivers completed the Persian version of the 7-point Samn-Perelli Fatigue Scale at the beginning and end of their shifts. They scored themselves as 1 (Fully alert, wide awake), 2 (Very lively, responsive, but not at peak), 3 (Okay, somewhat fresh), 4 (A little tired, less than fresh), 5 (Moderately tired, let down), 6 (Extremely tired, very difficult to concentrate), and 7 (Completely exhausted, unable to function effectively).^24,25^

 Considering that the Samn-Perelli scale can only measure overall fatigue, the Persian version of the Fatigue Assessment Scale (FAS) was utilized to assess the physical and mental dimensions that train drivers typically experience. The physical dimension included 3 items (physically, I feel exhausted, I get tired very quickly, and I am bothered by fatigue). In addition, the mental dimension contained 7 items (Mentally, I feel exhausted, I don’t do much during the day, I have problems thinking clearly, I have problems starting things, I have not enough energy for everyday life, and I feel no desire to do anything. When I am doing something, I can concentrate quite well). Train drivers were asked to select one out of five answer categories for each item, ranging from never to always (1 = never, 2 = sometimes, 3 = regularly, 4 = often, and 5 = always). The score for each dimension was calculated by summing all the items. A higher score indicates higher levels of fatigue.^26^ The validity and reliability of both the Persian version of the Samn-Perelli scale and the Persian version of the FAS have been confirmed in the current study and previous studies.^4,27^

###  Mental Workload

 The mental workload was evaluated using the Persian version of the National Aeronautics and Space Administration Task Load Index (NASA-TLX).^28^ The train drivers completed the NASA-TLX scale at the mid- and end-points of their shifts. It is a self-assessment scale confirmed as the most widely used workload measure,^28,29^ including in the railway industries.^14^ This questionnaire includes mental workload, physical workload, time pressure, performance, effort, and frustration dimensions. Each dimension is scored on a scale of 0–100. The scores for each dimension were entered separately and then combined for the overall score in the statistical analysis. To calculate the overall score, we followed the procedures outlined by Hart and Staveland^30^ and Rubio et al.^31^ First, the drivers were asked to select the member of each pair dimension that provided the most significant source of workload. Next, they were asked to mark the scale of each dimension. Finally, the overall score was determined using the following equation:


$$Overall\;score=\frac{\sum \left(Rating\;for\;each\;dimension\times Weight\;of\;dimension\right)}{15}$$

A higher score indicated a higher level of mental workload received. The validity and reliability of the Persian version of the NASA-TLX have been confirmed in the current study and previous studies.^4,27^

###  Statistical Analysis

 Descriptive statistics were conducted on the data (including frequencies, means, and standard deviations). The Spearman test was used to explore the correlation between fatigue and mental workload. The Pearson test determined the correlations among physical fatigue, mental fatigue, and mental workload. The paired t-test was utilized to compare the mean value of fatigue and mental workload at the beginning and mid-end points of shifts. Further, multiple linear regressions were applied to evaluate the mental workload effects on fatigue with consideration of age, sleep duration, work experience, and the like. The data were analyzed by the IBM SPSS software (version 21) and the GraphPad PRISM software (version 5). The significance level of statistical tests was defined as 0.05 (5%) or 0.001 (1‰).

## Results

**Figure 1:**
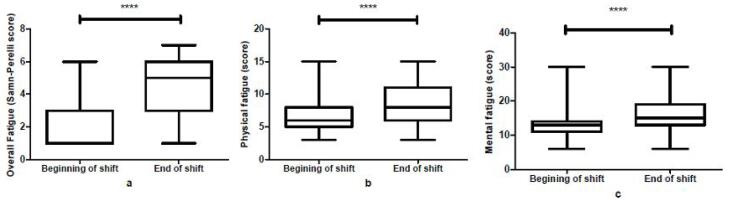
Overall fatigue (a), physical fatigue (b), and mental fatigue (c) of train drivers (N = 863) at different times of their shifts. *Note*. Values are means ± standard deviation; ^****^P < 0.001

 Based on the results (Table 1), the participants were young (29.73 ± 4) and educated (about 86%) and had relatively low experience (6.18 ± 3.34). The demographic characteristics of drivers showed that the sleep duration of participants was normal (7.42 ± 3.44), and most of them worked day shifts (about 95.5%). In addition, having a second job and smoking were not prevalent. The results of overall fatigue indicated that 50.1% of participants reported fatigue levels as “Fully alert, wide awake” at the beginning of shift work. However, more than 30% of participants demonstrated a high degree of fatigue at the end of the shift. The results of overall fatigue, mental fatigue, and physical fatigue of train drivers at the beginning and end of shifts are shown in Figure 1. The overall fatigue at the end of the shift was higher than its level at the beginning of the shift (Figure 1a). A significant difference (*P*< 0.001) was found between these two times. Furthermore, the physical fatigue at the end of the shift was higher than its level at the beginning of the shift, and a significant difference (*P*< 0.001) was observed between these two times (Figure 1b). The mental fatigue at the end of the shift was higher than its level at the beginning of the shift, representing a significant difference (*P*< 0.001) between these two times (Figure 1c).

**Figure 2:**
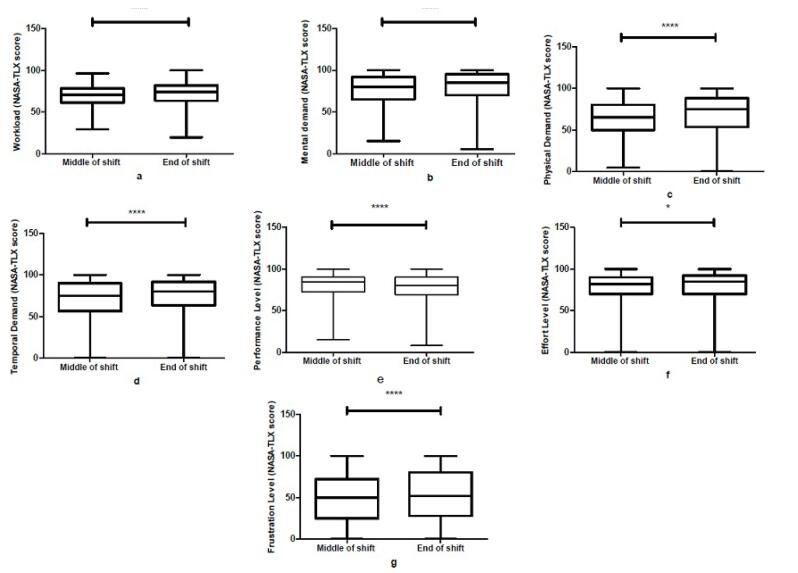
Mental workload of train drivers (N = 863) at different times of their shifts. *Note*. Values are means ± standard deviations; ^*^P< 0.05,^****^P< 0.001

 The comparison of subjective mental workload (based on the NASA-TLX) demonstrated that the overall workload (Figure 2a) and its dimensions, including the mental (Figure 2b), physical (Figure 2c), temporal (Figure 2d), and performance (Figure 2e) demands, as well as the effort (Figure 2f) and frustration (Figure 2g) levels, were significantly increased at the end of shift than at the middle of shift. Most of these increases were strongly significant between these two times (*P*< 0.001).

Table 2 provides a correlation between workload and fatigue dimensions among 863 train drivers. Based on the data in Table 3, there was the strongest correlation between overall workload and time pressure (R = 0.68, *P*< 0.001). The next important dimensions were physical (R = 0.66, *P*< 0.001) and mental (R = 0.63, *P*< 0.001) workloads, respectively. Overall fatigue among train drivers had a maximum correlation with time pressure (R = 0.23, *P*< 0.001) and physical workload (R = 0.20, *P*< 0.001), respectively. Overall workload was more affected by physical fatigue (R = 0.39, *P*< 0.001) than mental fatigue (R = 0.31, *P*< 0.001). Frustration had the maximum correlation with physical (R = 0.38, *P*< 0.001) and mental (R = 0.34, *P*< 0.001) fatigue. The results of linear regression showed a significant correlation between overall workload and overall (*P*< 0.001), physical (*P*< 0.001), and mental (*P* < 0.001) fatigue, without considering confounding factors.

**Table 2:** The correlation coefficient between fatigue and workload dimensions among train drivers (N = 863)

	**Overall workload**	**Mental workload**	**Physical workload**	**Time pressure**	**Performance**	**Effort**	**Frustration**	**Overall fatigue**	**Physical fatigue**
Mental fatigue	0.15	0.04	0.07	0.12	-0.13	-0.03	0.34	0.31	0.64
Physical fatigue	0.16	0.03	0.10	0.14	-0.14	-0.02	0.38	0.39	
Overall fatigue	0.27	0.15	0.20	0.23	-0.13	-0.04	0.14		
Frustration	0.60	0.18	0.31	0.30	-0.07	0.12			
Effort	0.53	0.33	0.24	0.29	0.28				
Performance	0.35	0.22	0.13	0.21					
Time constraints	0.68	0.49	0.4						
Physical workload	0.66	0.43							
Mental workload	0.63								

 The results of Table 3 revealed a significant correlation between overall fatigue and overall workload (*P*< 0.001). In addition, there were significant correlations between overall fatigue and work experience (*P*= 0.020), overtime (*P*< 0.001), and work hours (*P*= 0.010). Based on the findings, a significant correlation was observed between physical fatigue and overall workload (*P*< 0.001). Likewise, there were significant correlations between physical fatigue with smoking (*P*= 0.008), shift type (*P*= 0.020), and work hours a week (*P*= 0.001). Moreover, a significant correlation was found between mental fatigue and overall workload (*P*< 0.001). Further, mental fatigue with smoking (*P*= 0.010) and work hours (*P* < 0.001) demonstrated significant correlations.

**Table 3:** Multiple linear regression between overall workload and fatigue dimensions, with control of confounding factors among train drivers (N = 863)

**Variables**	**Overall fatigue**			**Physical fatigue**			**Mental fatigue**		
	**Estimate**	**Standard error**	***P *** **value**	**Estimate**	**Standard error**	***P *** **value**	**Estimate**	**Standard error**	***P *** **value**
Constant	0.857	1.317	0.515	1.756	2.420	0.046	8.305	3.747	0.027
Workload	0.004	0.001	0.001	0.010	0.002	0.001	0.015	0.002	0.001
Age	0.059	0.033	0.077	0.090	0.062	0.148	0.125	0.095	0.188
Night sleep	-0.010	0.052	0.849	0.002	0.094	0.981	-0.099	0.147	0.499
Second job	0.095	0.290	0.744	-0.031	0.537	0.562	-0.659	0.846	0.436
Work experience	-0.071	0.031	0.021	-0.069	0.056	0.216	-0.156	0.086	0.071
Education level	0.178	0.100	0.075	0.357	0.192	0.064	0.083	0.298	0.779
Home to work distance	0.052	0.137	0.706	0.222	0.257	0.388	0.204	0.399	0.609
Smoking	-0.404	0.251	0.108	-1.235	0.462	0.008	-1.689	0.716	0.019
Shift type	0.156	0.118	0.186	0.508	0.218	0.020	0.562	0.340	0.098
Overtime a week	0.046	0.011	0.001	0.034	0.023	0.145	0.030	0.036	0.408
Work hours a week	0.015	0.006	0.014	0.038	0.012	0.001	0.064	0.018	0.001

## Discussion

 Human cognitive abilities and limitations play an important role in the majority of industrial accidents and driving crashes.^7-9^ Fatigue, especially mental one, is generally inevitable for employees. Fatigue usually results in work-related damage, decreased productivity, performance loss, and critical human errors.^10^ Therefore, management of mental fatigue is essential from the point of view of controlling work risk, productivity, and job well-being. This study was designed to evaluate fatigue and its correlation to mental workload in train drivers. At the onset of shift work, about 1.7% of train drivers reported a high level of fatigue. However, 32.6% of drivers indicated moderate to high fatigue at the end of shift work. Based on the literature, fatigue and its impairment can be measured on a 7-point scale (completely exhausted) according to the Samn-Perelli scale.^25^ Therefore, 32.8% of train drivers (about 288) were prone to accidents at the end of shift work. Jay et al reported the same results and indicated a significant increase in fatigue based on the Semn-Perelli scale among the train drivers at the post-trip in comparison to the pre-trip baseline for all shifts.^32^ Furthermore, Kazemi et al, by using the same scale, reported the fatigue of train drivers at the beginning and end of a trip. There was a significant difference between these two times.^21^ Using the multidimensional fatigue instrument, Tsao et al concluded that train driver fatigue was significantly influenced by feelings related to working overtime.^14^ This finding confirmed that train driving is one of the most critical jobs in modern transportation.

 The results of this study showed that mental fatigue among train drivers was more important than physical fatigue at the end of the shift. In addition, the mental workload dimension was the most important workload problem at the end of the shift. Baysari et al underlined that almost all 40 railway accidents investigated in Australia were associated with mental workload.^17^ Dunn and Williamson indicated that nearly all train drivers found their job to be fatiguing and/or tiring and roughly half of these drivers reported experiencing fatigue and/or tiredness at least half of the shifts that they worked. Additionally, they underlined that the highest and lowest scores were related to mental and physical demands, respectively, based on the NASA-TLX.^33^ Dorrian et al^25^ and Kazemi et al^21^ achieved the same results for the conventional train drivers of the Australian and Iranian Rail Industries. Stone et al found high levels of mental tiredness in UK train drivers, respectively.^34^ In a study of Chinese railways, the workload was assessed by the NASA-TLX index, and the results highlighted that mental demand was rated as having the highest score among all dimensions by the train drivers.^14^ Concerning fatigue and NASA-TLX scores, a close correlation can be observed between the results of this study and other studies in different countries. The mental workload seems to be an integral part of a train-driving job and may be related to the monitoring and vigilance tasks of train drivers. Zoer et al, in a systematic literature search, published that the psychological work characteristics of train drivers include mental tasks, psychological demands, and decision latitude.^35^ High cognitive demands in the train-driving job can cause a high level of mental workload.

 The results of this study demonstrated a complex correlation between workload and overall, mental, and physical fatigue. However, the analysis of NASA-TLX domains exhibited a high and positive correlation between the overall workload and the time pressure, the physical workload, the mental workload, and the frustration. The overall fatigue had a maximum positive correlation with the time pressure, the physical workload, the mental workload, and the frustration. In addition, frustration was found to be significantly and positively correlated with mental fatigue and physical fatigue. Furthermore, the time pressure had a significant correlation with mental fatigue and physical fatigue. According to past studies, mental demand, time pressure, and frustration are associated with vigilance and stress.^36,37^ Additionally, past studies introduced mental fatigue and physical fatigue as a result of stress.^38,39^ Therefore, it seems that stress is the key factor in drivers’ fatigue. The stress may result in time pressure, mental workload, physical workload, and frustration for drivers. In other words, sustained attention (vigilance) and the physical demand of driving tasks are the central causes of stress. Although mental fatigue had no significant correlation with the mental and physical workloads, and physical fatigue had no significant correlation with the mental workload, a high correlation formed between mental fatigue and physical fatigue showed the same source. This means that stress, as a source of fatigue, may affect mental fatigue and physical fatigue.

 Multiple linear regression demonstrated a significant correlation between overall workload, overall fatigue, mental fatigue, and physical fatigue with and without control of confounding factors. These results confirm the direct effect of workload on fatigue. The same correlation was reported in the current study, which is in line with the findings of Dorrian et al; they introduced sleep length, shift duration, and workload rating as the predictors of extreme tiredness/exhaustion.^25^ Moreover, previous studies indicated that workload, shift duration, lifestyle, age, and shift start time are causal factors among the drivers.^11^ The current study found no significant correlation between overall fatigue, physical fatigue, and mental fatigue with the night shift, educational level, home-to-work distance, and second job. However, Dorrian et al declared that the proper average sleep length (7.2 hours) and shift duration (8 hours) are generally considered acceptable from a fatigue perspective.^25^ In addition, there are facilities to rest for drivers. After working for about one and a half hours, they can rest for about half an hour, so these resting times can be helpful for recovery and refreshing.

 Several limitations must be considered in this large, cross-sectional study. First, due to the limited resting time of drivers, there were restrictions on the instructions given to train drivers for completing the questionnaires. Second, considering the number of subjects (n = 1194) and the potential for interference in their job performance, we utilized subjective tools to assess fatigue and mental workload rather than objective tools. It is recommended that dynamic analysis be employed to investigate the interaction effect of significant variables obtained in these studies over time. Additionally, it is suggested that qualitative studies be conducted to develop intervention programs aimed at reducing the fatigue and workload of metro operation drivers based on current and past findings.

**Highlights**

- A cross-sectional study was conducted to evaluate mental workload and work fatigue in metro train operations.
- There was a significant difference in overall, physical, and mental fatigue at the middle and end of the shift.
- Mental workload and its dimensions were significantly higher at the end of the shift than in the middle of the shift.
- The mental dimension was the most important workload problem among the train drivers.
- Overall workload and fatigue had a maximum correlation with time pressure.

## Conclusion

 The results obtained from the current study confirmed that the fatigue level increases significantly at the end of the shift, and so train drivers are more prone to accidents during these times. Moreover, the train drivers experience different levels of mental workload, consequently resulting in fatigue. The mental workload had a significant correlation with the work fatigue of the train drivers. Control measures should be focused on the mental workload and related dimensions, especially mental demand and time pressure. In addition to the workload, different factors can cause fatigue. Fatigue can be affected by age, lifestyle, work experience, overtime, and work hours. Further intervention programs should be focused on this concern. The complex human-machine system imposes many mental workloads on the operators. On the other hand, the mental workload can result in physical and mental fatigue. Ultimately, fatigue can reduce operators’ health and productivity, and mental fatigue can be considered one of the leading causes of human errors and accidents in the complex human-machine system.

## Acknowledgments

 The research team would like to thank Tehran Metro for its cooperation and all participants in this study. They would also like to thank the Farzanegan Nik Andish Institute for the statistical analyses.

## Authors’ Contribution

**Conceptualization:** Yahya Khosravi, Narmin Hassanzadeh-Rangi, Ali-Asghar Farshad.

**Data curation:** Narmin Hassanzadeh-Rangi, Yahya Khosravi, Hamed Jalilian.

**Formal analysis:** Narmin Hassanzadeh-Rangi, Yahya Khosravi.

**Funding acquisition:** Ali-Asghar Farshad.

**Investigation:** Yahya Khosravi, Narmin Hassanzadeh-Rangi.

**Methodology:** Yahya Khosravi, Narmin Hassanzadeh-Rangi, Ali-Asghar Farshad.

**Project administration:** Yahya Khosravi, Ali-Asghar Farshad.

**Resources:** Ali-Asghar Farshad.

**Software:** Narmin Hassanzadeh-Rangi, Yahya Khosravi.

**Supervision:** Yahya Khosravi, Ali-Asghar Farshad.

**Validation:** Yahya Khosravi, Narmin Hassanzadeh-Rangi.

**Visualization:** Narmin Hassanzadeh-Rangi, Yahya Khosravi.

**Writing–original draft:** Narmin Hassanzadeh-Rangi, Hamed Jalilian, Yahya Khosravi.

**Writing–review & editing:** Narmin Hassanzadeh-Rangi, Hamed Jalilian, Yahya Khosravi, Ali-Asghar Farshad.

## Competing Interests

 The authors declare no conflict of interests to claim.

## Ethical Approval

 All study methods, including human participation, followed the guidelines and regulations approved by the Iran University of Medical Sciences. These guidlines are based on the general principles of ethics in medical science research in the Islamic Republic of Iran and the Declaration of Helsinki. Written informed consent forms were obtained from all participants before they took part in this study.

## Funding

 This work was supported by Tehran Metro through a research contract with the Occupational Health Research Center at Iran University of Medical Sciences.
